# Hepatocyte-Specific Deficiency of DAX-1 Protects Mice from Acetaminophen-Induced Hepatotoxicity by Activating NRF2 Signaling

**DOI:** 10.3390/ijms231911786

**Published:** 2022-10-04

**Authors:** Young-Joo Suh, Hyo-Jeong Yun, Yu-Bin Kim, Eun-Jung Kang, Jung Hyeon Choi, Young-Keun Choi, In-Bok Lee, Dong-Hee Choi, Yun Jeong Seo, Jung-Ran Noh, Jong-Soo Lee, Yong-Hoon Kim, Chul-Ho Lee

**Affiliations:** 1 Laboratory Animal Resource Center, Korea Research Institute of Bioscience and Biotechnology (KRIBB), Daejeon 34141, Korea; 2 College of Veterinary Medicine, Chungnam National University, Daejeon 34134, Korea; 3 Department of Functional Genomics, KRIBB School of Bioscience, University of Science and Technology (UST), Daejeon 34113, Korea

**Keywords:** DAX-1, acetaminophen, Nrf2, hepatotoxicity

## Abstract

Acetaminophen (APAP) is a widely used analgesic and antipyretic drug, but its overdose can cause acute liver failure. The dosage-sensitive sex reversal adrenal hypoplasia congenita critical region on the X chromosome, gene 1 (DAX-1, NR0B1), is an orphan nuclear receptor that acts as a transcriptional co-repressor of various genes. In this study, we identified the role of DAX-1 in APAP-induced liver injury using hepatocyte-specific *Dax-1* knockout (*Dax-1* LKO) mice. Mouse primary hepatocytes were used as a comparative *in vitro* study. APAP overdose led to decreased plasma alanine aminotransferase and aspartate aminotransferase levels in *Dax-1* LKO mice compared to C57BL/6J (WT) controls, accompanied by reduced liver necrosis. The expression of the genes encoding the enzymes catalyzing glutathione (GSH) synthesis and metabolism and antioxidant enzymes was increased in the livers of APAP-treated *Dax-1* LKO mice. The rapid recovery of GSH levels in the mitochondrial fraction of APAP-treated *Dax-1* LKO mice led to reduced reactive oxygen species levels, resulting in the inhibition of the prolonged JNK activation. The hepatocyte-specific DAX-1 deficiency increased the protein expression of nuclear factor erythroid 2-related factor 2 (Nrf2) compared with WT controls after APAP administration. These results indicate that DAX-1 deficiency in hepatocytes protects against APAP-induced liver injury by Nrf2-regulated antioxidant defense.

## 1. Introduction

Acetaminophen (APAP) is a commonly used analgesic and antipyretic drug but an overdose leads to severe hepatotoxicity, which is the major cause of acute liver failure globally [1]. In particular, in the United States, 50,000 people visit the emergency department and over 500 deaths are recorded annually due to APAP overdose [1,2]. At a therapeutic dosage, most APAP is metabolized by phase II conjugating enzymes and excreted in urine. Some of it forms N-acetyl-p-benzoquinone imine (NAPQI), a highly reactive intermediate metabolite, through cytochrome P450 enzymes and is detoxified via glutathione (GSH) conjugation [3]. However, excessive NAPQI due to APAP overdose depletes GSH and binds to mitochondrial proteins [4]. This triggers mitochondrial oxidative stress, which is considered to be the predominant cellular event in APAP-induced hepatotoxicity [3]. In addition, it causes the excessive production of reactive oxygen species (ROS) and activation of c-Jun N-terminal kinase (JNK) signaling [5]. Sustained JNK activation ultimately results in liver cellular destruction and necrosis [6]. Despite years of research on APAP hepatotoxicity, the pathophysiological process of APAP overdose is not well understood and the therapeutic options are significantly limited.

The nuclear factor erythroid 2-related factor 2 (Nrf2) is a transcription factor that plays a critical role in cellular detoxification following oxidative stress. Once Nrf2 is activated, Nrf2-mediated gene expression comprising GSH synthesis and phase II detoxifying enzymes, such as glutathione S-transferase alpha 1 (GSTA1) and NAD(P)H: quinone oxidoreductase 1 (NQO1) is turned on [7]. Nrf2 activity is primarily regulated by its natural repressor protein, Kelch-like ECH-associated protein 1 (Keap1), which directly causes the proteasomal degradation of Nrf2 [8]. Nrf2 signaling is related to many types of liver diseases, including APAP-induced liver injury [9,10].

The dosage-sensitive sex reversal adrenal hypoplasia congenita critical region on the X chromosome, gene 1 (DAX-1, NR0B1), is an orphan nuclear receptor (NR), which lacks a known endogenous receptor ligand [11]. DAX-1 acts as a transcriptional co-repressor of other NRs and plays a key role in mammalian gonad development and sex determination [12]. DAX-1 is most closely related to the *NR0B2* encoding small heterodimer partner (SHP) and both share considerable structural and functional similarities, such as the repressive effect of gluconeogenesis and lipogenesis in the liver [13,14]. According to previous studies, hepatocyte-specific SHP deficiency alleviated APAP-induced hepatotoxicity by inhibiting the MKK4-JNK pathway through growth arrest and DNA damage-inducible 45 beta (GADD45β) regulation [15]. In addition, *Shp*-deficient mice were more susceptible to concanavalin A-induced fulminant hepatitis and endotoxin-induced septic shock indicating that SHP might control the immune response [16,17]. However, relatively few research studies have focused on the role of DAX-1 in APAP-induced hepatotoxicity. This study examined the role of DAX-1 in APAP-evoked liver injury and investigated the underlying mechanism.

## 2. Results

### 2.1. DAX-1 Deficiency Ameliorates APAP-Induced Hepatocellular Damage in Primary Hepatocytes

**Figure 1:**
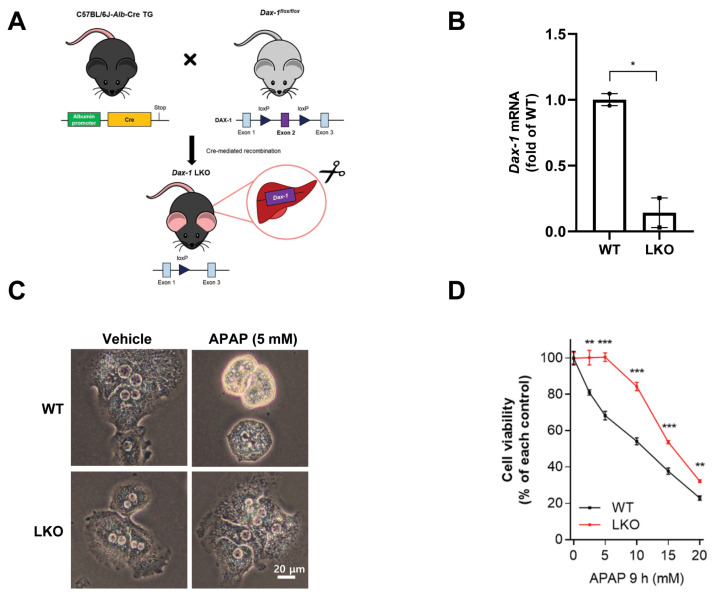
DAX-1 deficient hepatocytes are tolerant towards APAP-induced cell death. (**A**,**B**) Generation and validation of hepatocyte-specific *Dax-1* deletion mice. (**A**) Schematic diagram of the mouse strains used in the study. *Alb*-Cre mice were crossed to the line of mice carrying the floxed *Dax-1* sequences to delete *Dax-1* in the hepatocytes. LoxP sites were introduced on either side of exon 2, resulting in deletion of exon 2. (**B**) Primary hepatocytes were isolated from C57BL/6J (WT) and *Dax-1* LKO mice. Gene expression levels of *Dax-1* were analyzed by quantitative real-time PCR (RT-qPCR reactions were conducted in duplicate). The mRNA levels were normalized by 18S rRNA and shown as the fold change relative to the WT group (set as 1). Two-tailed Student’s *t*-test was used to compare the WT group with the LKO group. * *p* < 0.05. (**C**,**D**) Primary mouse hepatocytes isolated from WT and *Dax-1* LKO mice were treated with indicated concentrations of APAP for 9 h. (**C**) Representative images of mouse primary hepatocyte morphology. (**D**) Cell viability was determined using a colorimetric cell viability assay kit. Data were obtained from three independent experiments. All data are expressed as mean ± SEM. Two-tailed Student’s *t*-test was used to compare the WT group at each time point with the LKO group. ** *p* < 0.01 and *** *p* < 0.001.

*Dax-1* LKO mice were obtained by crossing *Dax-1^flox/flox^* mice with C57BL/6J-*Alb*-Cre TG mice (Figure 1A). To assess the clearance efficacy of DAX-1 in the hepatocytes, the *Dax-1* mRNA level was measured via RT-qPCR, and the levels for C57BL/6J (WT) and *Dax-1* LKO mice were compared. The *Dax-1* mRNA level decreased in the primary hepatocytes of *Dax-1* LKO mice (Figure 1B). Next, to investigate the hepatocyte-specific role of DAX-1 in APAP-induced hepatotoxicity, primary hepatocytes obtained from WT or *Dax-1* LKO mice were incubated in up to 20 mM APAP for 9 h. Morphological changes were observed in WT primary hepatocytes after APAP treatment. Most of the *Dax-1* LKO hepatocytes remained attached to the culture plate, whereas WT hepatocytes aggregated to form clusters and detached from the plate (Figure 1C). APAP-induced cell death was significantly reduced in *Dax-1* LKO hepatocytes compared with WT hepatocytes (Figure 1D). These results indicate that DAX-1 deficiency in hepatocytes offers protective effects against APAP hepatotoxicity.

### 2.2. Hepatocyte-Specific Dax-1 Deficient Mice Are More Resistant to APAP-Induced Hepatotoxicity

**Figure 2:**
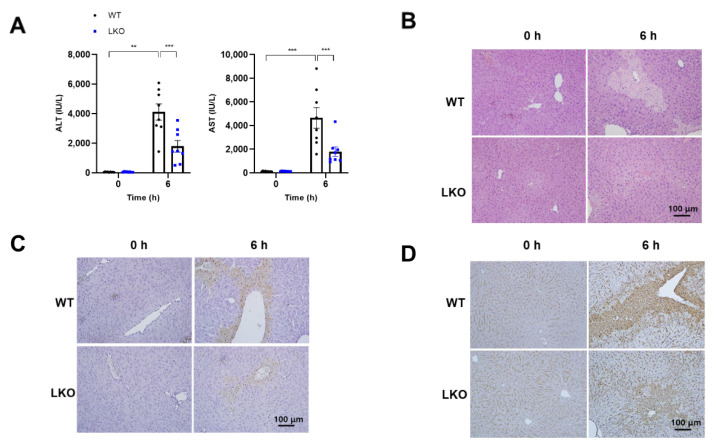
Hepatocyte-specific *Dax-1* LKO mice show high resistance to APAP-induced hepatotoxicity. WT and *Dax-1* LKO mice were subjected to intraperitoneal injection with APAP (300 mg/kg) or water. (**A**) Plasma ALT and AST activities were measured at 0 (*n* = 8–9) and 6 h (*n* = 8) after APAP administration. Representative images of (**B**) H&E staining, (**C**) TUNEL assay, and (**D**) immunohistochemistry staining for nitrotyrosine of liver tissues harvested 0 and 6 h after APAP treatment. Data are expressed as mean ± SEM. ** *p* < 0.01 and *** *p* < 0.001 (Tukey–Kramer test after the one-way ANOVA).

As shown in Figure 1, DAX-1 deficient hepatocytes were more resistant to APAP-induced toxicity. To ascertain the hepatocyte-specific role of DAX-1 in APAP-induced hepatotoxicity, WT and *Dax-1* LKO mice were intraperitoneally injected with a single dose of APAP (300 mg/kg). The most critical time points for investigating any signaling events involved in APAP-induced liver injury are 6 h after APAP overdose in mice [18]. Therefore, we first confirmed whether there was a difference in lesions between WT and *Dax-1* LKO mice at 6 h after APAP treatment. As expected, APAP induced severe liver injury at 6 h as indicated by the elevated plasma alanine aminotransferase (ALT) and aspartate aminotransferase (AST) levels, and extensive parenchymal necrosis in the liver (Figure 2). However, this increase was noticeably reduced in *Dax-1* LKO mice. DAX-1 deficiency attenuated the increase in plasma ALT and AST levels and reduced the necrotic area, as observed through hematoxylin and eosin (H&E) staining (Figure 2A,B). The extent of the damaged hepatocyte was confirmed using the terminal deoxynucleotidyl transferase dUTP nick end labeling (TUNEL) assay and nitrotyrosine immunohistochemistry, indicators of DNA fragmentation and peroxynitrite formation, respectively. WT mice showed apparent hepatocyte injury, which was substantially reduced in the livers of *Dax-1* LKO mice (Figure 2C,D), suggesting that hepatocyte-specific DAX-1 deficiency can potentially protect against APAP-induced liver injury.

### 2.3. APAP-Induced Mitochondrial Oxidative Stress Is Attenuated by Hepatocyte-Specific DAX-1 Deficiency in Mice

**Figure 3:**
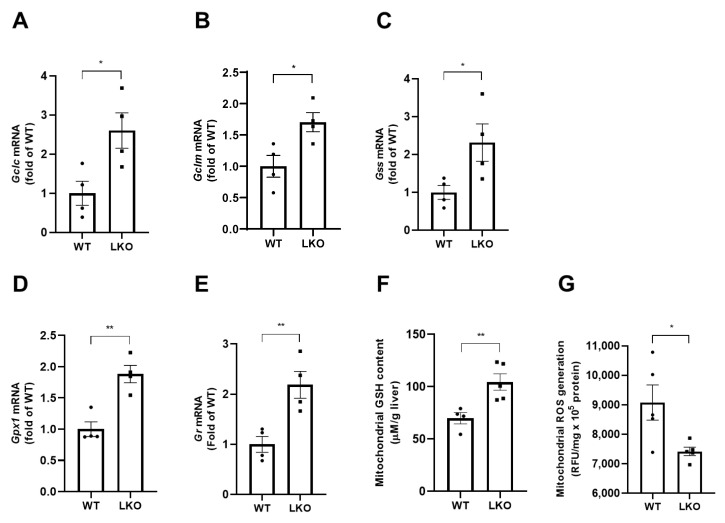
DAX-1 deficiency in hepatocytes attenuates APAP-induced mitochondrial GSH depletion and ROS production. Liver tissues from WT and *Dax-1* LKO mice were harvested at 6 h (*n* = 4–5) after APAP (300 mg/kg) treatment. Gene expression levels associated with GSH synthesis (**A**–**C**) and GSH metabolism (**D**,**E**) were analyzed by quantitative real-time PCR. The mRNA levels were normalized by 18S rRNA and shown as the fold change relative to the WT group (set as 1). (**F**) Mitochondrial GSH levels were measured by enzymatic analysis. (**G**) Total reactive oxygen species (ROS) production in mitochondrial fractions of the liver was measured with the DCFHDA assay. Data are expressed as mean ± SEM. Two-tailed Student’s *t*-test was used to compare the WT group with the LKO group. * *p* < 0.05 and ** *p* < 0.01.

To determine whether the attenuation of APAP-induced liver damage in *Dax-1* LKO mice was caused by altered APAP metabolism, we examined the protein expression level of the major APAP metabolizing enzyme in the liver at 6 h after APAP treatment. The protein levels of Cyp2e1, a primary enzyme responsible for the conversion of APAP to its toxic metabolites [19], in WT and *Dax-1* LKO mice were comparable (Supplementary Figure S1). These results demonstrate that the protective effects of DAX-1 deficiency are independent of APAP metabolism regulation. Next, we examined the expression of GSH-related genes, which play a pivotal role in the detoxification of APAP [20]. Gene expression of glutamate-cysteine ligase catalytic subunit (*Gclc*), glutamate-cysteine ligase modifier subunit (*Gclm*), and glutathione synthetase (*Gss*), related to GSH synthesis, and glutathione reductase (*Gr*) and glutathione peroxidase 1 (*Gpx1*), related to GSH metabolism, was significantly increased in *Dax-1* LKO mice compared to APAP-treated WT mice (Figure 3A–E). Moreover, mitochondrial GSH remained high in *Dax-1* LKO mice when compared to WT 6 h post-APAP administration (Figure 3F), along with a significant reduction in ROS levels (Figure 3G). Taken together, hepatocyte-specific DAX-1 deficiency decreased mitochondrial ROS level with an augmented GSH level.

### 2.4. Hepatocyte-Specific DAX-1 Deficiency Inhibits Persistent JNK Phosphorylation in the Liver of APAP-Treated Mice

**Figure 4:**
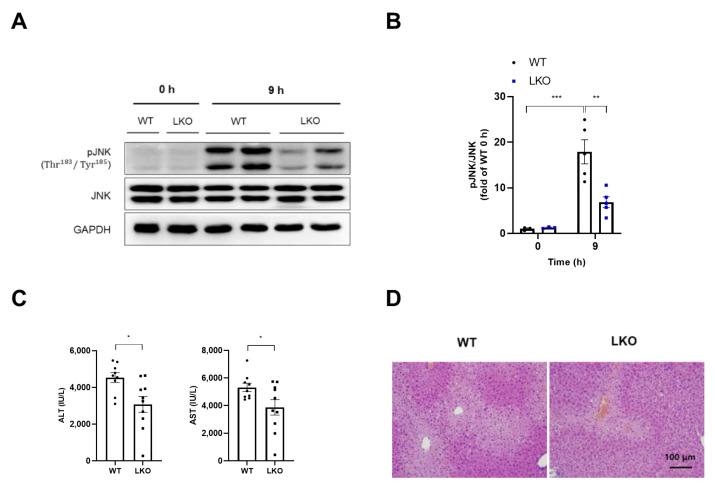
DAX-1 deficiency in hepatocytes reduces prolonged JNK phosphorylation mediated by APAP in the liver. (**A**,**B**) Liver tissues from WT and *Dax-1* LKO mice were harvested at 0 h (*n* = 3) and 9 h (*n* = 5) after APAP (300 mg/kg) treatment. (**A**) Western blot was used to detect phosphorylation of JNK. Total JNK and GAPDH were used as loading controls. (**B**) The graph shows the results of densitometric analysis of phospho-JNK relative to JNK and indicated as the fold change relative to the 0 h WT group (set as 1). All data are expressed as mean ± SEM. ** *p* < 0.01 and *** *p* < 0.001 (Tukey–Kramer test after the one-way ANOVA). (**C**) Plasma was collected at 9 h (*n* = 9–10) after APAP administration. Plasma ALT and AST levels were measured. (**D**) Representative images of H&E staining in liver tissues 9 h after APAP treatment. Two-tailed Student’s *t*-test was used to compare the WT group with the LKO group. * *p* < 0.05.

Prolonged JNK induction in hepatocytes plays an important role in acute liver injury, which leads to cell death [21]. JNK activation was confirmed at 9 h because LKO is thought to have alleviated sustained JNK activation, which is associated with advanced mitochondrial GSH recovery and reduced ROS levels. The present study found that the administration of APAP significantly induced JNK phosphorylation in the liver of WT mice after 9 h, but was markedly attenuated in *Dax-1* LKO mice (Figure 4A,B). Furthermore, APAP treatment in WT mice showed considerable liver damage after 9 h, as revealed by increased ALT and AST plasma levels, and further severe centrilobular necrosis indicated by H&E staining (Figure 4C,D). These results demonstrate that DAX-1 deficiency continues to protect against hepatotoxicity after APAP administration and blocks sustained JNK activation.

### 2.5. DAX-1 Deficiency in Hepatocytes Enhances the Expression of Genes Encoding Antioxidant Enzymes in the Liver following APAP Treatment

**Figure 5:**
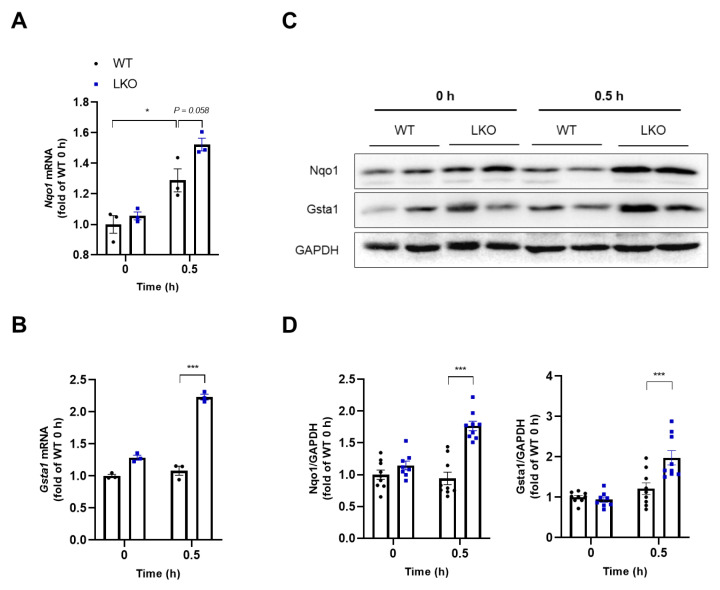
DAX-1 deficiency in hepatocytes increases expression levels of antioxidant genes and proteins following APAP treatment in the liver. Liver tissues from WT and *Dax-1* LKO mice were harvested at 0 (*n* = 8–9) and 0.5 h (*n* = 9) after APAP (300 mg/kg) treatment. The total RNA was extracted and the transcriptional levels of (**A**) *Nqo1* and (**B**) *Gsta1* were determined by RT-qPCR (three technical replicates for each pooled sample from mice). The mRNA levels were normalized by 18S rRNA and shown as the fold change relative to the 0 h WT group (set as 1). (**C**) The protein levels of Nqo1 and Gsta1 were determined using western blot analysis. GAPDH was used as the loading control. (**D**) Relative protein was quantitatively expressed by densitometric analysis and shown as the fold change relative to the 0 h WT group (set as 1). Data are expressed as mean ± SEM. * *p* < 0.05 and *** *p* < 0.001 (Tukey–Kramer test after the one-way ANOVA).

Total GSH amount is limited in the mouse liver, and it is rapidly depleted within the first 0.5 h after an APAP overdose [18]. Therefore, we investigated the level of GSH depletion 0.5 h after APAP treatment, and there was no difference between WT and *Dax-1* LKO mice (Supplementary Figure S2). The increase in oxidative stress due to excessive ROS accumulation is one of the hallmarks of APAP-induced liver injury [22]; therefore, the ROS scavenging system is important. As the roles of antioxidant enzymes are critical, we determined the expression levels of Nqo1 and Gsta1. Although there was no difference between the basal expression levels of transcripts and proteins of Nqo1 and Gsta1 in WT and *Dax-1* LKO mice, they were significantly increased in *Dax-1* LKO mice compared with WT mice after 0.5 h of APAP treatment (Figure 5). These results suggest that DAX-1 deficiency in hepatocytes appears to be important in reducing oxidative stress by improving the antioxidant system after APAP administration.

### 2.6. Hepatocyte-Specific DAX-1 Deficiency Increases Nrf2 Expression in the Liver of Mice Induced by APAP

**Figure 6:**
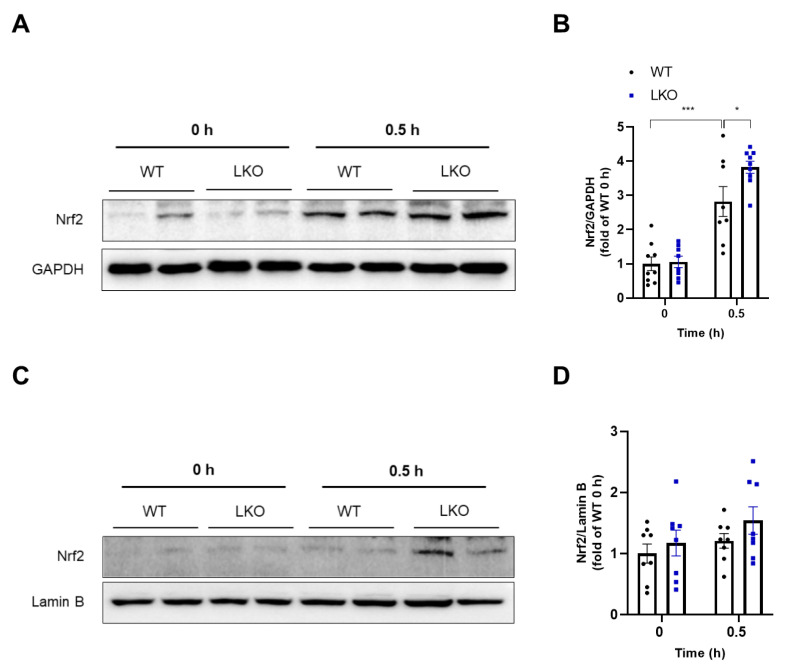
Hepatocyte-specific DAX-1 deficiency activates antioxidant response by regulating Nrf2 protein levels in the liver of APAP-treated mice. (**A**,**B**) Liver tissues from WT and *Dax-1* LKO mice were harvested at 0 and 0.5 h (*n* = 8–9) after APAP (300 mg/kg) treatment. (**A**) The protein levels were determined using western blot analysis. GAPDH was used as the loading control. (**B**) Relative protein was quantitatively expressed by densitometric analysis. (**C**,**D**) Liver nuclear extracts from WT and *Dax-1* LKO mice were prepared at 0 and 0.5 h after APAP treatment. (**C**) The protein levels of nuclear Nrf2 were determined using western blot analysis. Lamin B was used as the loading control. (**D**) Relative protein was quantitatively expressed by densitometric analysis and shown as the fold change relative to the 0 h WT group (set as 1). Data are expressed as mean ± SEM. * *p* < 0.05 and *** *p* < 0.001 (Tukey–Kramer test after the one-way ANOVA).

Next, we investigated the upstream factors regulating the expression of GSH-related and antioxidant genes as illustrated in Figure 3 and Figure 5. According to previous studies, these are all target genes of Nrf2, an important antioxidant transcription factor, and Nrf2 activation is required to protect against APAP-induced liver injury [23,24]. Furthermore, as shown in Supplementary Figure S2, the substantial depletion of GSH can activate the Nrf2 pathway [25]. Therefore, we examined whether DAX-1 deficiency can activate Nrf2 in the livers of APAP-treated mice at early time points. Compared to WT mice, Nrf2 protein levels were significantly increased 0.5 h after APAP treatment in *Dax-1* LKO mice (Figure 6A,B). Furthermore, APAP-treated *Dax-1* LKO mice tended to induce nuclear translocation of Nrf2 (Figure 6C,D). DAX-1 is a nuclear receptor that mainly controls the expression of other genes [12]. Therefore, to confirm whether the increase in Nrf2 protein levels in *Dax-1* LKO mice was related to nuclear receptor-mediated transcriptional repression, we examined the gene expression of *Nrf2*. In contrast with western analysis data, *Nrf2* mRNA levels in WT and *Dax-1* LKO mice were comparable (Supplementary Figure S3). These results suggest that the elevation of Nrf2 protein level in *Dax-1* LKO mice is not attributed to transcriptional regulation. We also investigated the mechanism of Nrf2 activation by confirming the expression of Keap1, which plays a central role in the regulation of Nrf2 activity [26]. However, there were no significant differences between WT and Dax-1 LKO mice in both western and RT-qPCR analyses (Supplementary Figure S4). Taken together, the protein expression of Nrf2 was enhanced by the deletion of DAX-1, which may provide a possible mechanism for DAX-1 to alleviate APAP-induced hepatotoxicity.

## 3. Discussion

In many countries, APAP overdose is the leading cause of acute liver failure [27]. The hepatocyte-specific deletion of SHP ameliorates APAP-induced hepatotoxicity [15]. Although DAX-1 is expressed at lower levels in the liver compared to SHP, it plays an important role in repressing diverse nuclear receptors such as hepatocyte nuclear factor 4α, constitutive androstane receptor, liver X receptor α, and farnesoid X receptor [13,28]. Because hepatocytes are the major cell types involved in APAP-induced liver injury [29], the present study used hepatocyte-specific *Dax-1* deletion mice to demonstrate whether DAX-1 deficiency can modulate APAP-induced hepatotoxicity.

After the administration of the toxic doses of APAP, *Dax-1* LKO mice exhibited reduced overall hepatotoxicity, including decreased plasma ALT and AST levels and attenuated levels of histopathological liver damage. Intriguingly, this phenomenon was observed in primary murine hepatocytes and mice liver, indicating a hepatocyte-specific role of DAX-1 in modulating APAP-induced liver injury. APAP overdose elevated GADD45β expression, which is related to the inhibition of the MKK4-JNK pathway, and *Shp* knockout mice increased it [15,30]. Therefore, there is a high possibility that DAX-1 inhibits GADD45β expression, as the related NR0B family member, SHP, showed in previous results [15]. We assessed the mRNA levels of GADD45β and did not observe relevant changes (Supplementary Figure S5A,C). Herein, we hypothesize that DAX-1 inhibits SHP, which regulates GADD45β, based on previous studies suggesting that DAX-1 acts as a corepressor through interactions with other NRs [31]. However, DAX-1 deficiency did not affect APAP-induced SHP expression (Supplementary Figure S5B,D). Therefore, we suggest that the regulatory mechanism involved in DAX-1 deficiency after APAP administration differs from the one in the induction of GADD45β expression.

The main events of APAP hepatotoxicity are toxic metabolite generation by Cyp2e1, depletion of hepatic GSH, and formation of APAP-protein adducts [32]. Based on this mechanistic insight, *N*-acetylcysteine, which is the only currently approved antidote against APAP overdose, focuses on the replenishment of hepatic GSH [33]. In the current study, DAX-1 deficiency had no effect on Cyp2e1-mediated reactive metabolite formation. Furthermore, WT and *Dax-1* LKO mice showed similar GSH depletion after 0.5 h, an early time point in APAP administration. As the expression of the GSH-related genes increased 6 h after APAP treatment, these results suggest that DAX-1 plays a role in modulating APAP hepatotoxicity through the recovery of GSH, independent of APAP metabolism.

Nrf2 is a key molecule that plays a protective role in APAP-induced liver injury by regulating antioxidant-responsive element-mediated gene expression and increasing GSH synthesis [23]. Upon exposure to oxidative stress, Nrf2 translocates to the nucleus to induce its target genes [34]. We elucidated the effect of DAX-1 on the Nrf2 signaling pathway as the expression of Nrf2 target genes *Gclc*, *Gclm*, *Gss*, *Gr*, *Gpx1*, *Nqo1*, and *Gsta1* upregulated. We confirmed that Nrf2 protein levels were increased in *Dax-1* LKO mice within a short time after APAP treatment. In addition, although the Nrf2 protein level in the nucleus of *Dax-1* LKO mice was slightly increased after 0.5 h of APAP administration, the expression of well-established Nrf2 regulated genes Nqo1 and Gsta1 was significantly upregulated, suggesting that DAX-1 deficiency enhanced Nrf2 transactivation. Because DAX-1 is known as a transcriptional repressor [13], the observed increase in Nrf2 protein level upon APAP injection may be mediated by its function. However, DAX-1 deficiency did not elevate APAP-induced Nrf2 gene expression, indicating that DAX-1 did not influence Nrf2 transcription. In the present study, we demonstrated that the loss of DAX-1 in hepatocytes can positively regulate the post-translational modification of Nrf2, thus guarding against APAP-induced hepatotoxicity. This provided insight into the mechanism of DAX-1 in susceptibility to APAP toxicity. Moreover, Nrf2 protein levels increase after oxidative stress because its negative regulator, Keap1, forfeits its ability to bind to Nrf2 [35]. Lately, the direct disruption of Keap1–Nrf2 protein–protein interaction has been identified as a promising method for improving Nrf2 activity [36]. The activation of Nrf2 signaling in the present study may be due to the reduced expression of the Keap1 protein in the presence of APAP. We therefore investigated whether the regulation of Nrf2 activity by DAX-1 was related to Keap1. However, Keap1 expression was not affected, indicating that DAX-1 does not directly regulate Keap1. A detailed mechanism for the Nrf2 regulation of DAX-1 should be proposed; our studies suggest the important roles of DAX-1 in mediating Nrf2 activation.

Although JNK is an important component of the stress response, prolonged JNK activation plays a crucial role in APAP-induced liver damage [6]. The initial JNK activation is triggered by the early mitochondrial stress caused by mitochondrial protein adduct formation [37,38]. Although early JNK activation does not necessarily result in severe downstream damage, sustained JNK activation is directly related to liver injury [39]. Therefore, a JNK-mitochondria signaling loop, which indicates that the amplified oxidant stress in mitochondria sustains cytosolic JNK activation, is important [37]. In the current study, ROS decreased, whereas the GSH level in mitochondria of *Dax-1* LKO mice increased after 6 h of APAP administration. However, JNK phosphorylation drastically decreased in *Dax-1* LKO mice after 9 h instead of 6 h after APAP treatment (Supplementary Figure S6). As ROS generated from mitochondria are conceivable second messengers in activating JNK, this is one possible explanation for the subsequent inhibition of JNK activity. We suggest that Nrf2 upregulation in *Dax-1* LKO mice increased the expression of GSH synthesizing genes as well as antioxidant genes, and these enzymes enhanced the recovery of mitochondrial GSH levels, which increased the scavenging capacity for reactive oxygen in the liver after the metabolism of APAP was complete. In turn, reduced mitochondrial ROS attenuates prolonged JNK activation, thereby reducing hepatocyte death. Evidence for this mechanism is obtained from previous studies where APAP-treated female mice showed low susceptibility by eventually attenuating late JNK activation through accelerated mitochondrial GSH recovery and enhanced ROS detoxification [40]. These results indicate that the deletion of DAX-1 affects this prolonged JNK activation by enhancing the subsequent GSH recovery and inhibiting hepatic mitochondrial oxidative stress, which is related to Nrf2-mediated antioxidative signaling pathways.

In terms of the approaches for DAX-1 regulation, only a small portion of DAX-1 inhibition strategies are known. MicroRNA, as one of them, has been shown to regulate DAX-1 expression. It was previously reported that microRNA-181 promotes prostate cancer by directly inhibiting DAX-1 [41]. As microRNA-181 is highly expressed in the liver [42], targeting microRNA is expected to protect against APAP-induced liver injury by inhibiting DAX-1. However, further research is still needed in this regard.

The role of DAX-1 in APAP hepatotoxicity was confirmed in hepatocytes, which are parenchymal cells, but it was also necessary to confirm in hepatic non-parenchymal cells. Myeloid cells are known to play an important role in responding to tissue damage in the early stage of acetaminophen-induced liver injury by releasing several cytokines and chemokines [43]. Therefore, we examined whether DAX-1 deletion in hepatic non-parenchymal cells influences APAP-induced liver injury using *Dax-1* myeloid cell-specific knockout (MKO) mice. Blood analysis and H&E staining of the liver were performed 6 h after APAP administration to WT and *Dax-1* MKO mice, but there was no significant difference between the two groups (Supplementary Figure S7). These results indicate that DAX-1 plays an independent role in the direct regulation of myeloid cells in APAP-induced liver injury. Thus, we believe that this study is more meaningful because it firstly elucidates the cell type-specific role of DAX-1 in APAP hepatotoxicity particularly in hepatic parenchymal cells.

In summary, our findings demonstrate that hepatocyte-specific DAX-1 deficiency protects against APAP overdose-induced hepatotoxicity via enhanced Nrf2-regulated antioxidant defense. We therefore propose the loss of DAX-1 as a potential Nrf2 activator and the therapeutic target for liver injury caused by APAP overdose. However, the relationship between the hepatoprotective effect of DAX-1 deficiency and the activation of Nrf2 requires further investigation. The challenges related to APAP hepatotoxicity treatment might be partially overcome by DAX-1 targeting strategies.

## 4. Materials and Methods

### 4.1. Animals

Male C57BL/6J wild-type (WT) mice were obtained from the Korea Research Institute of Bioscience and Biotechnology (KRIBB; Daejeon, Korea). C57BL/6J-*Alb*-Cre transgenic (TG) mice, C57BL/6J-*Lyz*-Cre transgenic (TG) mice, and C57BL/6J mice containing floxed alleles for exon 2 of the *Dax-1* gene *Dax-1^flox/flox^* were obtained from Jackson Laboratory (Bar Harbor, ME, USA). To create mice with hepatocyte-specific DAX-1 deletion (*Dax-1* LKO) and myeloid cell-specific DAX-1 deletion (*Dax-1* MKO), *Dax-1^flox/flox^* mice were crossed with C57BL/6J-*Alb*-Cre TG mice and C57BL/6J-*Lyz*-Cre TG mice, respectively. Mice were kept in a specific pathogen-free facility with a 12 h light/dark cycle at 22 ± 2 °C. All animals were accustomed to the experimental room for 1 week with free access to food and water. Prior to APAP (Sigma-Aldrich Chemical Co., St. Louis, MO, USA) administration, mice were fasted for 16 h, but with free access to water. APAP was dissolved in warm distilled water (55–60 °C) and cooled to 37 °C before it was intraperitoneally injected into the mice.

### 4.2. Isolation of Primary Mouse Hepatocytes and In Vitro APAP Treatment

Primary mouse hepatocytes were isolated from mice by perfusion with collagenase type I, as previously described [15]. Subsequently, primary hepatocytes were seeded onto 24-well culture dishes for cell viability tests. The cells were cultured overnight under monolayer conditions in low glucose Dulbecco’s modified Eagle’s medium (WelGENE Inc., Gyeongsan-si, Gyeongsangbuk-do, Korea) containing 10% fetal bovine serum, 100 units/mL penicillin, and 100 μg/mL streptomycin, in a humidified environment (5% CO_2_) at 37 °C, followed by treatment with APAP. After the final 9 h of culture with APAP, cell viability was measured using the Cell Counting Kit-8 (Dojindo Molecular Technology, Inc., Rockville, MD, USA) with 2-(2-methoxy-4-nitrophenyl)-3-(4-nitrophenyl)-5-(2,4-disulfophenyl)-2H-tetrazolium monosodium salt (WST-8).

### 4.3. Blood Analysis

Plasma alanine aminotransferase (ALT) and aspartate aminotransferase (AST) activities were determined using an automated blood chemistry analyzer (AU480; Beckman Coulter, Krefeld, Germany).

### 4.4. Histopathology and Immunohistochemistry

Liver tissues fixed in 10% neutral buffered formalin were embedded in paraffin, cut into 5-μm-thick sections, and stained with H&E for necrosis evaluation. Nitrotyrosine staining was performed to assess nitrotyrosine protein adducts, using an anti-nitrotyrosine antibody (Millipore Co., Billerica, MA, USA) and visualized with 3, 3′-diaminobenzidine (DAB; Vector Lab, Burlingame, CA, USA).

### 4.5. TUNEL Staining

TUNEL staining was performed for DNA strand break assessment using sections of paraffin-embedded tissue samples according to the manufacturer’s instructions (Chemicon International, Temecula, CA, USA). In this method, DNA strand breaks were deoxygenated by terminal deoxynucleotidyl transferase. DNA fragments were then labeled with digoxigenin, bound to anti-digoxigenin-peroxidase, and visualized via DAB.

### 4.6. Western Blot Analysis

Mouse livers were homogenized in ice-cold lysis buffer (0.1 mmol/L sodium vanadate, 1 mmol/L phenylmethanesulfonyl fluoride, 25 mmol/L NaF, 50 mmol/L Tris-HCl, 40 mmol/L β glycol phosphate, 120 mmol/L NaCl, 1% NP40, and 0.5% Triton X-100) containing protease inhibitor and phosphatase inhibitor. The homogenates were centrifuged thrice at 16,600× *g* for 10 min at 4 °C, and the protein concentration in the supernatant was measured using the Bradford assay. Protein and phosphorylation levels were analyzed by western blotting using anti-phospho-JNK, anti-JNK, anti-GAPDH (Cell Signaling Technology, Danvers, MA, USA), anti-Cyp2e1, anti-Nrf2, anti-Keap1, anti-Nqo1, and anti-Gsta1 antibodies (Abcam, Cambridge, MA, USA) following standard procedures. Proteins were quantified via densitometric analysis of the ImageJ software version 1.43u (Wayne Rasband National Institutes of Health, Bethesda, MA, USA).

### 4.7. RNA Extraction, Reverse Transcription, and RT-qPCR

Total RNA was isolated from the mouse livers using TRIzol reagent (Invitrogen, Waltham, Massachusetts, USA). cDNA was synthesized using the UltraScript 2.0 cDNA Synthesis Kit (PCR Biosystems, London, UK) according to the manufacturer’s instructions. Subsequently, qPCR was performed using AccuPower^®^ 2X Greenstar™ qPCR MasterMix (Bioneer, Daejeon, Korea) and the StepOne™ Real-time PCR system (Applied Biosystems, Foster City, CA, USA). RNA pooling was carried out by mixing the same amount of each cDNA sample at a fixed concentration in a single tube and diluting it 10-fold. Relative gene expression levels were analyzed using the 2^−ΔΔCt^ method and normalized to 18S rRNA. The primer sequences used in the present study are presented in Table S1.

### 4.8. Isolation of Subcellular Fractions

Liver tissues were homogenized with hypotonic buffer (0.5 M HEPES, 1 M MgCl_2_, 1 M KCl, 1 M DTT, and dH_2_O) containing the protease inhibitor. The tissue homogenates were centrifuged at 700× *g* for 10 min at 4 °C to pellet the nuclei. Nuclei were washed with hypotonic buffer and centrifuged again, and the pellet was resuspended in lysis buffer. The supernatant of homogenate was centrifuged at 5000× *g* for 10 min at 4 °C to precipitate the mitochondria. As with nuclear fractionation, the pellet was washed and centrifuged again. To determine the mitochondrial GSH, the mitochondrial pellet was resuspended in MES buffer (0.4 M 2-(N-morpholino) ethanesulphonic acid, 0.1 M phosphate, and 2 mM EDTA) and in lysis buffer for mitochondrial ROS production. Nuclei and mitochondria were sonicated and centrifuged at 16,600× *g* for 15 min at 4 °C.

### 4.9. Measurements of Liver GSH Levels

To determine total hepatic GSH contents and GSH in the mitochondrial liver fractions, liver tissues and isolated mitochondria from the liver were centrifuged, respectively, at 10,000× *g* for 15 min at 4 °C. The supernatant was deproteinated before the assay. GSH measurement was performed using a commercial kit (Cayman Chemical Company, Ann Arbor, MI, USA). The total GSH content was calculated based on the slope of the standard curve.

### 4.10. Measurements of Mitochondrial ROS Levels

The total levels of ROS were measured via the oxidative conversion of the nonfluorescent 2′,7′-dichloro-dihydro-fluorescein diacetate (DCFHDA; Invitrogen, Waltham, MA, USA) to the highly fluorescent 2′,7′-dichlorofluorescein (DCF) in the mitochondrial liver fractions. Liver extracts were incubated at 37 °C for 60 min with a 1 mM DCFHDA. Subsequently, fluorescence intensity was measured at 485 nm excitation and 530 nm emission using a SpectraMax iD5 Multi-Mode Microplate Reader (Molecular Devices, San Jose, CA, USA) and normalized to the protein content.

### 4.11. Statistical Analysis

All values were expressed as mean ± SEM. Student’s *t*-test was used when two independent groups were compared, and the Tukey–Kramer test after one-way ANOVA (GraphPad Prism v8.4.3, GraphPad Software, San Diego, CA, USA) extended the *t*-test to more than two groups. All statistical tests with *p* < 0.05 were regarded as statistically significant.

## Data Availability

The data that support the findings of this study are available from the corresponding author upon reasonable request.

## Supplementary Materials

The following supporting information can be downloaded at: https://www.mdpi.com/article/10.3390/ijms231911786/s1.
