# Supplementary material for: Hepatocyte-Specific Deficiency of DAX-1 Protects Mice from Acetaminophen-Induced Hepatotoxicity by Activating NRF2 Signaling
Source: Int J Mol Sci. 2022 Oct 4;23(19):11786. doi: 10.3390/ijms231911786 (PMC9570285; doi:10.3390/ijms231911786)
Supplement: Supplementary file 1 [file ijms-23-11786-s001.zip › ijms-1923130-supplementary.pdf]

1. Supplementary figures and legends

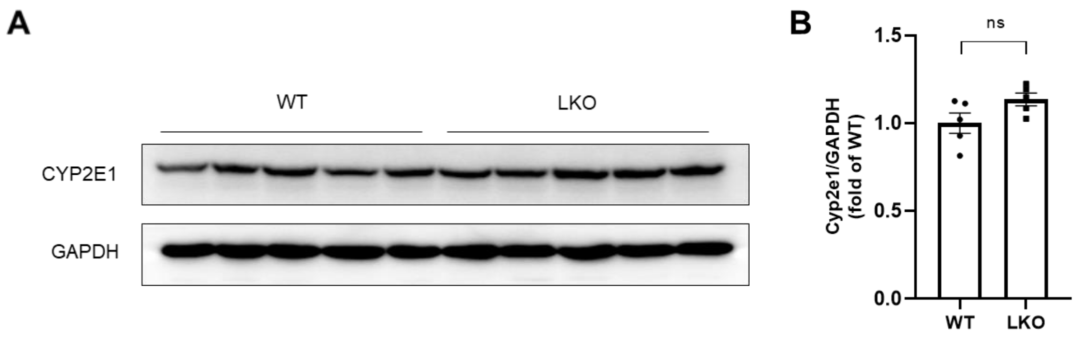

Supplementary Figure S1. DAX-1 deficiency does not alter Cyp2e1 protein levels. Liver tissues from WT and *Dax-1* LKO mice were harvested at 6 h (n = 5) after APAP (300 mg/kg) treatment. (A) The protein levels of Cyp2e1 were determined using western blot analysis. GAPDH was used as the loading control. (B) The graph shows the result of densitometric analysis of Cyp2e1 relative to GAPDH. Data for the WT group was set as 1 and the average fold change is shown. Data are expressed as mean ± SEM. Two-tailed Student's *t*-test was used to compare the WT group with the LKO group. ns = no significant difference.

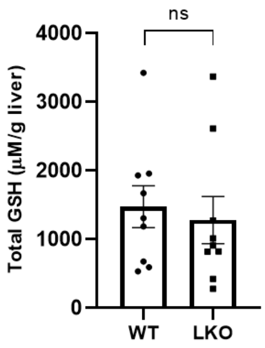

Supplementary Figure S2. DAX-1 deficiency in hepatocytes does not affect GSH depletion induced by APAP. Liver tissues from WT and *Dax-1* LKO mice were harvested at 0.5 h (n = 9) after APAP (300 mg/kg) treatment and total hepatic GSH content was measured by enzymatic analysis. Data are expressed as mean ± SEM. Two-tailed Student's *t*-test was used to compare the WT group with the LKO group. ns = no significant difference.

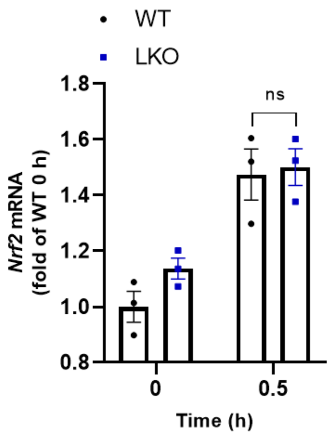

Supplementary Figure S3. Loss of DAX-1 in hepatocytes does not affect the expression of the *Nrf2* gene. The total RNA was extracted and the transcriptional levels of *Nrf2* were determined by quantitative real-time PCR (three technical replicates for each pooled sample from mice). The mRNA levels were normalized by 18S rRNA and the graph was shown as the fold change relative to the 0 h WT group (set as 1). Data are expressed as mean ± SEM. ns = no significant difference. (Tukey-Kramer test after the one-way ANOVA).

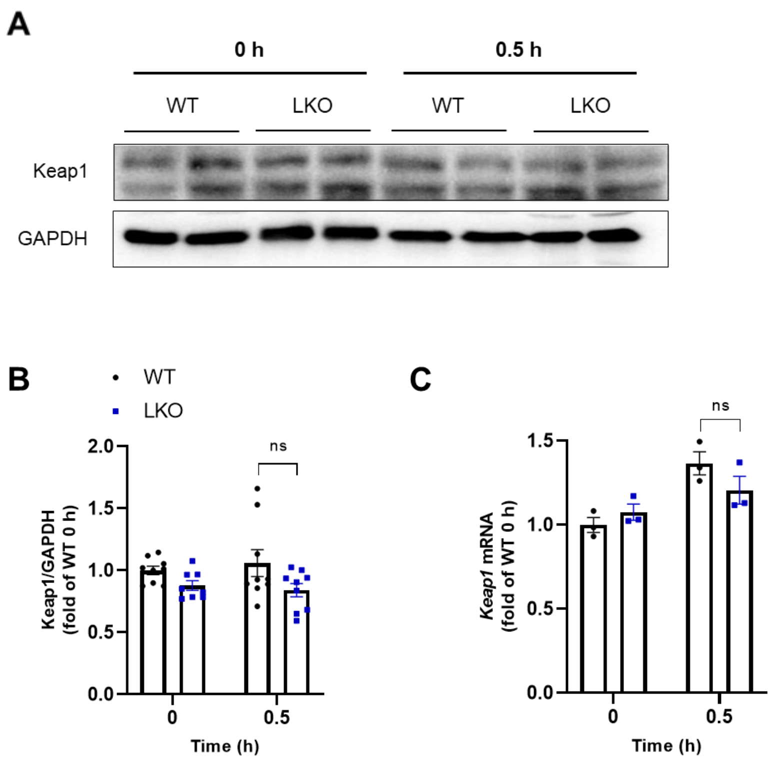

Supplementary Figure S4. Loss of DAX-1 in hepatocytes does not affect the expression of the Keap1 protein. Liver tissues from WT and *Dax-1* LKO mice were harvested at 0 (n = 8-9) and 0.5 h (n = 9) after APAP (300 mg/kg) treatment. The protein levels of (A) Keap1 were determined using western blot analysis. GAPDH was used as the loading control. (B) Relative protein was quantitatively expressed by densitometric analysis. The total RNA was extracted and the transcriptional levels of (C) Keap1 were determined by quantitative real-time PCR (three technical replicates for each pooled sample from mice). The mRNA levels were normalized by 18S rRNA and all graphs were shown as the fold change relative to the 0 h WT group (set as 1). Data are expressed as mean ± SEM. ns = no significant difference (Tukey-Kramer test after the one-way ANOVA).

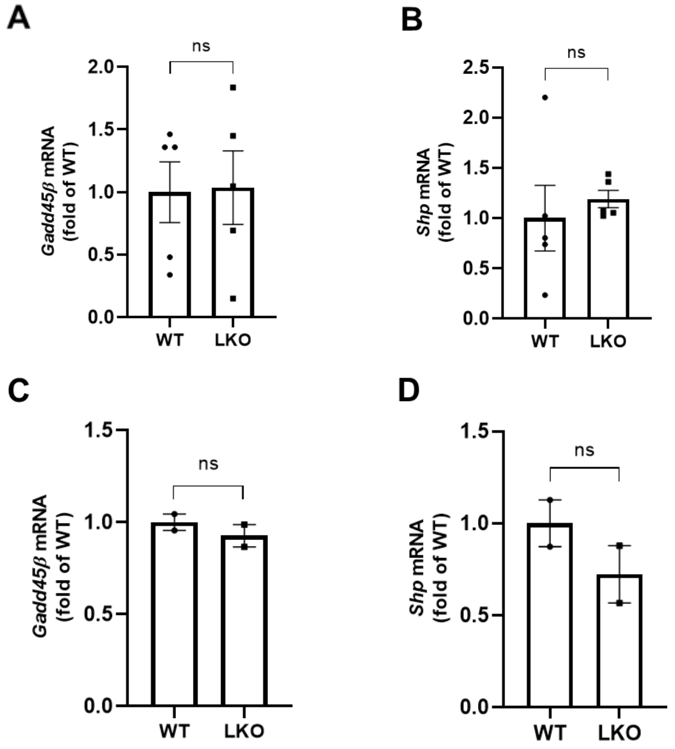

Supplementary Figure S5. Hepatocyte-specific DAX-1 deficiency does not correlate with GADD45β and SHP levels. (A, B) Liver tissues from WT and *Dax-1* LKO mice were harvested at 6 h (n = 5) after APAP (300 mg/kg) treatment and (C, D) primary hepatocytes isolated from WT and *Dax-1* LKO mice were treated with 10 mM of APAP for 9 h (RT-qPCR reactions in primary hepatocytes were conducted in duplicate). Gene expression levels of (A, C) *Gadd45β* and (B, D) *Shp* were analyzed by quantitative real-time PCR in both livers and primary hepatocytes. The mRNA levels were normalized by 18S rRNA and shown as the fold change relative to the WT group (set as 1). Data are expressed as mean ± SEM. Two-tailed Student's *t*-test was used to compare the WT group with the LKO group. ns = no significant difference.

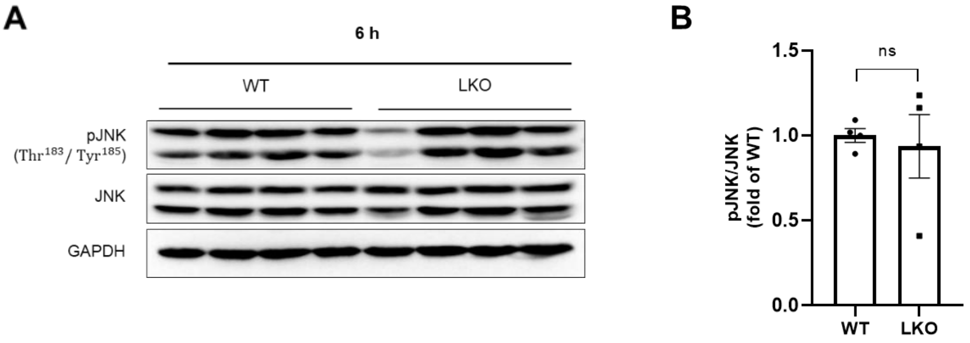

Supplementary Figure S6. No difference in JNK phosphorylation could be seen in the earlier time of the APAP-treated mice. Liver tissues from WT and *Dax-1* LKO mice were harvested at 6 h (n = 4) after APAP (300 mg/kg) treatment. (A) The JNK phosphorylation was evaluated by western blotting. Total JNK and GAPDH were used as loading controls. (B) The graph shows the results of densitometric analysis of phospho-JNK relative to JNK and indicated as the fold change relative to the WT group (set as 1). Data are expressed as mean ± SEM. Two-tailed Student's *t*-test was used to compare the WT group with the LKO group. ns = no significant difference.

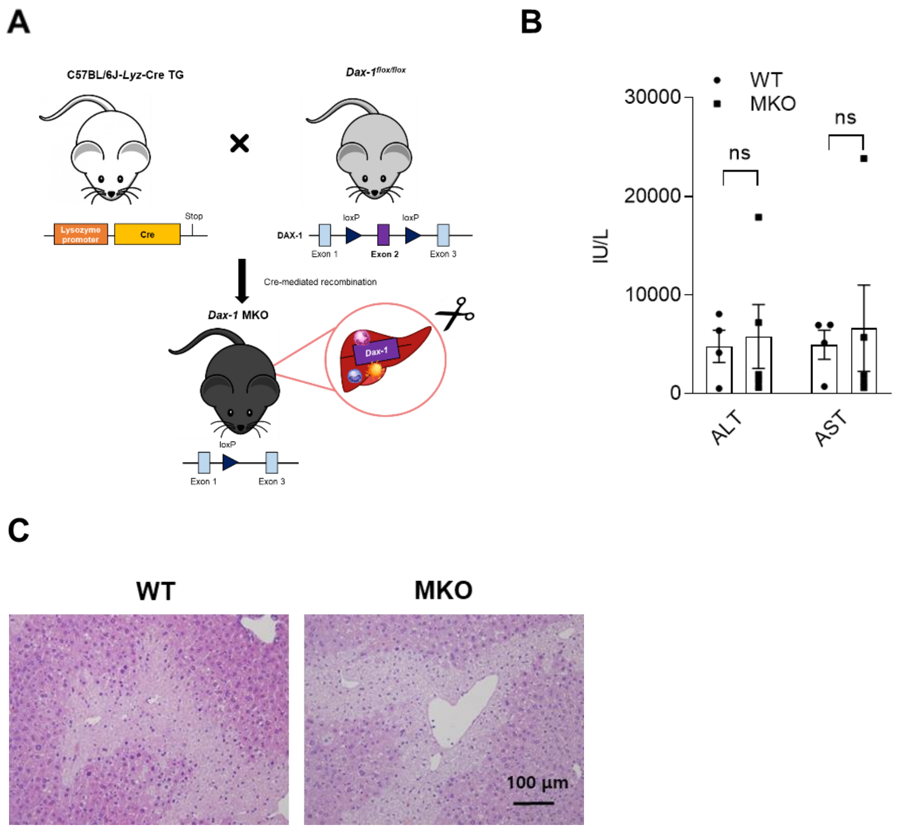

Supplementary Figure S7. Myeloid cell-specific *Dax-1* knockout mice (MKO) show no difference in APAP-induced liver toxicity. (A) Schematic diagram of the mouse strains used in the study. *Lyz*-Cre mice were crossed to the line of mice carrying the floxed *Dax-1* sequences, to delete *Dax-1* in the myeloid cells. LoxP sites were introduced on either side of exon 2, resulting in deletion of exon 2. (B, C) Liver tissues from WT and *Dax-1* MKO mice were harvested at 6 h (n = 4-5) after APAP (300 mg/kg) treatment. (B) Plasma ALT and AST levels were measured. Two-tailed Student's *t*-test was used to compare the WT group with the MKO group. ns = no significant difference (C) Representative images of H&E staining.

2. Supplementary table

Table S1. Sequences of PCR primers used in this study

Gene	Gene Bank Accession Number	Primer Sequence
Gclc	NM_010295.2	Forward 5′- TGGCTTTGAGTGCTGCATCT -3′<br>Reverse 5′- ATCACTCCCCAGCGACAATC -3′
Gclm	NM_008129.4	Forward 5′- TCACAATGACCCGAAAGAACTG -3′<br>Reverse 5′- ACCCAATCCTGGGCTTCAAT -3′
Gss	NM_001291111.1	Forward 5′- CAAAGCCTGGGAGCTCTATGG -3′<br>Reverse 5′- ACGGCACGCTGGTCAAATAT -3′
Gr	NM_010344.4	Forward 5′- TCCAAGTGGTGACTTCCGTG -3′<br>Reverse 5′- ATGTGGCCCTTTTCATCCGT -3′
Gpx1	NM_001329527.1	Forward 5′- TCCGGGACTACACCGAGATG -3′<br>Reverse 5′- TTCTCCTGGTGTCCGAACTGA -3′
Nqo1	NM_008706.5	Forward 5′- GGTTTACAGCATTGGCCACACT -3′<br>Reverse 5′- AACAGGCTGCTTGGAGCAAA -3′
Gsta1	NM_008181.3	Forward 5′- CGCCACCAAATATGACCTCT -3′<br>Reverse 5′- CCTGTTGCCCACAAGGTAGT -3′
Nrf2	NM_010902.5	Forward 5′- CCAGCTACTCCCAGGTTGC -3′<br>Reverse 5′- CCTGATGAGGGGCAGTGA -3′
Keap1	NM_001110305.1	Forward 5′- CCCATGAGGCATCACCGTAG -3′<br>Reverse 5′- CATAGCCTCCGAGGACGTAG -3′
Dax-1	NM_007430.5	Forward 5′- AGATTCATCAATAGCGATGT -3′<br>Reverse 5′- AGCTACGACCGCTTTCTCCA -3′
Gadd45β	NM_008655.1	Forward 5′- CGGAGACATTGGGCACAAC -3′<br>Reverse 5′- CCTTGGCTTTTCCAGGAATCT -3′
Shp	NM_011850.3	Forward 5′- TCTGCAGGTCGTCCGACTATT -3′<br>Reverse 5′- TGTCTTGGCTAGGACATCCA -3′
